# HisCoM-PAGE: Hierarchical Structural Component Models for Pathway Analysis of Gene Expression Data

**DOI:** 10.3390/genes10110931

**Published:** 2019-11-14

**Authors:** Lydia Mok, Yongkang Kim, Sungyoung Lee, Sungkyoung Choi, Seungyeoun Lee, Jin-Young Jang, Taesung Park

**Affiliations:** 1 Interdisciplinary Program in Bioinformatics, Seoul National University, Seoul 08826, Korea; 2 Department of Statistics, Seoul National University, Seoul 08826, Korea; 3 Center for Precision Medicine, Seoul National University Hospital, Seoul 03080, Korea; 4 Department of Applied Mathematics, Hanyang University (ERICA), Ansan 15588, Korea; 5 Department of Mathematics and Statistics, Sejong University, Seoul 05006, Korea; 6 Department of Surgery, Seoul National University College of Medicine, Seoul 03080, Korea

**Keywords:** pathway analysis, survival phenotype, Hierarchical structured component model

## Abstract

Although there have been several analyses for identifying cancer-associated pathways, based on gene expression data, most of these are based on single pathway analyses, and thus do not consider correlations between pathways. In this paper, we propose a hierarchical structural component model for pathway analysis of gene expression data (HisCoM-PAGE), which accounts for the hierarchical structure of genes and pathways, as well as the correlations among pathways. Specifically, HisCoM-PAGE focuses on the survival phenotype and identifies its associated pathways. Moreover, its application to real biological data analysis of pancreatic cancer data demonstrated that HisCoM-PAGE could successfully identify pathways associated with pancreatic cancer prognosis. Simulation studies comparing the performance of HisCoM-PAGE with other competing methods such as Gene Set Enrichment Analysis (GSEA), Global Test, and Wald-type Test showed HisCoM-PAGE to have the highest power to detect causal pathways in most simulation scenarios.

## 1. Introduction

Over the past several decades, gene expression data analysis has been the most common approach to investigate human diseases at the RNA level [1,2]. By analyzing gene expression data, we can gain a better understanding of disease etiology and biological mechanisms [3]. Especially for cancer prognosis, genetic information can be very effective in improving prognosis prediction of patients, based only on clinical information [4].

Analyzing high-throughput gene expression data, at the pathway level, is very effective in two ways. Firstly, grouping thousands of genes by their respective pathways reduces complexity to just several hundred pathways. Secondly, identifying active pathways that differ between two conditions, such as normal and tumor tissues, can have more explanatory power than a simple list of differentially expressed genes (DEGs) [5,6]. While there have been several methods proposed for gene set analysis, they mainly focused on the binary phenotypes. There are only a few methods available for dealing with survival phenotypes [7,8].

Various cancer prognosis and survival analysis have been reported [9,10]. For example, pancreatic cancer has a very poor prognosis, compared to other cancers. At the time of diagnosis, fewer than 20% of pancreatic cancer patients can have surgery, and their postoperative 5-year survival rate is also significantly low [11]. Therefore, more accurate pancreatic cancer prognosis, and early detection, are needed.

To build a good prediction model, using gene expression data, for actual clinical practice and medical intervention, it is first necessary to identify genes (features) related to prognosis. Exploring the pathways to which genes belong can provide valuable biological interpretation and help screen out false-positive genes. In this study, we mainly focus on finding significant pathways that are relevant to the prognosis of pancreatic cancer. Through pathway analysis, our ultimate goal is to identify biological mechanisms that influence the prognosis of disease more clearly.

Since gene set enrichment analysis (GSEA) was proposed, which uses the Kolmogorov-Smirnov statistic for measuring differentially expressed gene sets, many other pathway-based methods have been developed [12]. Recently, SetRank was developed to reduce the false positive hits of the GSEA [13]. Unlike other types of phenotypes, however, there are only a few pathway-based methods available for survival phenotypes. For example, the gene set variation analysis (GSVA) method was proposed to handle survival times by estimating the variation of pathway activity over a sample population in an unsupervised way [14]. The global test was proposed for continuous and censored survival time, based on the score statistics from random effects of parameters for association measure [15,16]. Likewise, the Wald test was proposed for the survival phenotype by summarizing the association measure from the sum of coefficients from a survival regression model [17]. However, those previous pathway methods are single pathway analyses, so they do not take into account correlations between pathways, and the global test only considers correlations between gene expression values. The Wald test merely sums up the statistics from each gene, to obtain its pathway statistics, so it does not account for the correlation among pathways. Since some genes may belong to several pathways simultaneously, there is a need for accounting for this nature of genes and pathways.

To account for this issue, we previously developed our Pathway-based approach using HierArchical structure of collapsed Rare variant Of High-throughput sequencing data (PHARAOH) method for discovering rare variants by constructing a hierarchical model that consists of collapsed gene-level summaries and entire pathways [18]. PHARAOH is based on the generalized structural component analysis (GSCA) model [19]. Later, we developed our Hierarchical structured CoMponent analysis of miRNA-mRNA integration (HisCoM-mimi) method to integrate anti correlated expression of miRNA and mRNA. By extension of PHARAOH, HisCoM-mimi can also account for the biological relationships between a miRNA and target mRNAs [20]. Recently, we developed another extension, Hierarchical structural CoMponent analysis of Gene-Gene Interactions (HisCoM-GGI), representing a model that not only summarizes common variants into gene levels, but also considers interactions among common variants [21]. 

In this study, we developed a new pathway-based model for survival phenotypes, based on gene expression data, by taking advantage of our earlier hierarchical model, referred to as HisCoM-PAGE which represents Hierarchical structural Component Models for Pathway Analysis for Gene Expression data. As an extension of HisCoM-mimi, HisCoM-PAGE considers the biological context of gene and pathway hierarchies, in the form of structured components. HisCoM-PAGE collapses genes into the pathway in a structured form using latent variables. Unlike other methods analyzing one pathway at a time, HisCoM-PAGE analyze all pathways simultaneously by one model, which enables considering the correlation of all pathways by using a ridge penalty in parameter estimation. HisCoM-PAGE can also successfully examine the effects of individual genes within the pathways. 

Through simulation studies, we showed that HisCoM-PAGE performed well, compared to other existing pathway methods for survival phenotype. Application to real microarray data of pancreatic ductal adenocarcinoma (PDAC) patients from Seoul National University Hospital (SNUH) showed that HisCoM-PAGE could well identify prognosis-related pathways. HisCoM-PAGE is available at (http://statgen.snu.ac.kr/software/HisCom-PAGE/).

## 2. Materials and Methods 

### 2.1. Materials

#### 2.1.1. SNUH—PDAC Microarray Data

125 PDAC samples were collected by the department of Hepatobiliary and Pancreatic Surgery of SNUH and all human subject studies were approved by the Institutional Review Board of SNUH. The PDAC patients’ average age was 63.32 years, and the standard deviation was 10.064 years; 75 patients were male, and 50 were female. The median survival time was 25 months. In this dataset, mRNA expression levels were generated using Affymetrix (Santa Clara, CA, USA) HuGene 1.0 ST arrays. A total of 32,321 genes were normalized by the Robust Multi-array Average (RMA) method [22]. Of the total, 21,369 genes were annotated. In this analysis, we selected mRNAs whose expressional variances were ranked in the top 25 percentiles for analysis [23,24]. The detail clinical information is described in Table 1.

#### 2.1.2. The Cancer Genome Atlas - PDAC RNA-Seq Data

The RNA-seq data and the clinical data were downloaded from The Cancer Genome Atlas (TCGA) GDC portal (https://portal.gdc.cancer.gov) [25]. For the RNA-seq data, an Illumina HiSeq instrument (San Diego, CA, USA) was used for mRNA profiling. In the sample selection procedure, non-PDAC samples were removed [26]. Also, samples with a survival time less than 3 months were removed, since the cause of death may not be due to PDAC. As a result, we analyzed 124 PDAC patients, among which there were 61 female samples and 63 male samples. The median survival time was 598 days, and the censoring proportion was 41%. The average age was 64.56 years, and its standard deviation was 10.91.

For the preprocessing procedure of RNA-seq data, the following steps were applied to 56,716 genes annotated. The Relative Log Expression (RLE) normalization method was adopted to control the gene length bias. The RLE method was implemented in R package (v3.5) “DESeq2” (v1.22.2) [27]. After RLE normalization, the genes with zero proportion larger than 80% were filtered out [28]. The number of remaining genes was 37,405. Next, the RNA-seq mRNA expression data were log2 transformed. Using unsupervised filtering based on Median Absolute Deviation (MAD), 9,380 genes whose MADs were ranked in the top 25 percentiles were finally selected for analysis.

#### 2.1.3. Simulation Data

To evaluate the performance of the HisCoM-PAGE method and compare its performance with other pathway methods, we generated a simulation data set, following the simulation settings of Lee et al [29]. In the simulation study, the following parameters were considered: the sample size (*I*), total number of genes (*K*), pathway size (*m_s_*), proportion of censoring (*c_p_*), and the proportion of significant genes in the pathway (*m_p_*). Gene expression data were randomly generated from a multivariate normal distribution with mean zero and covariance matrix Σ. Let the **O** zero matrix be *l × (K-l)* dimensions, where *l* is the number of causal genes within the gene set. Let ***I****_l_* be an *l × l* identity matrix, and ***A*** be a *l × l* symmetric matrix. Then, the covariance matrix is given as follows:

$$\underset{K\times K}{\sum }\;=\;\left[\begin{array}{cc} A & O\\ O^{T} & 0{.2I}_{K-l} \end{array}\right]$$

Four different scenarios of covariance matrix Σ were considered. For each scenario, ***A*** has a different structure. Here, *i,j* represent each row and column index for covariance matrix. For Scenario 1, A = 0.2**I***_l_*; for Scenario 2, **A** = 0.2[*x_ij_*] and *x_ij_* = 0.02; for Scenario 3, **A** = 0.2[*x_ij_*], and *x_ij_* = 0.1^|*i−j*|^. Scenario 4 has random variances and covariance, such that **A** is given as follows: **A** = 0.2[*x_ij_*], *x_ij_* = *ρ_ij_*, when *i* is not equal to *j*, and 1, when *i* is equal to *j,* and *ρ_ij_* is generated from N(0,0.1^2^). For all scenarios, three different significant gene proportion was considered. The survival time was generated from a Cox model with a constant baseline hazard rate of 0.005 whereas the censoring time was generated from an exponential distribution with a parameter of λ whose value depends on the censoring fraction [30]. The survival time and censoring time were generated independently. We only observed the minimum value of either the survival time or the censoring time, which occurred first. For power analysis, the regression coefficients w were generated from the uniform distribution *U*(0.2,0.6). For type 1 error estimation, the regression coefficient w was assumed to be zero. 

### 2.2. Methods

#### 2.2.1. HisCoM-PAGE Method

**Figure 1:**
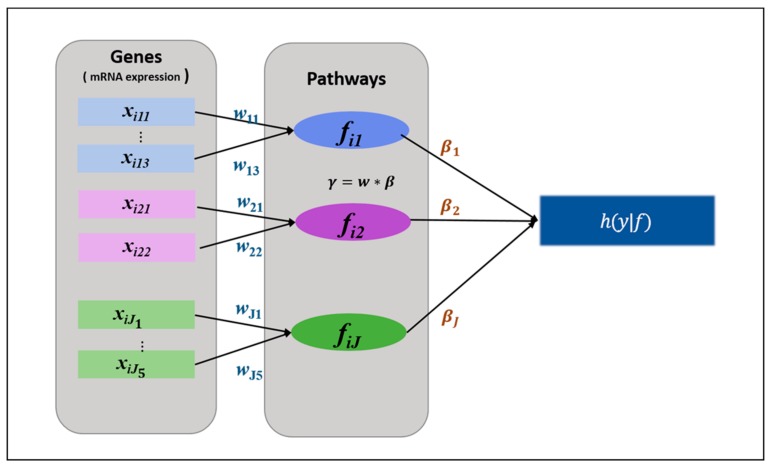
A schematic diagram of the HisCoM-PAGE model. Figure 1 shows the HisCoM-PAGE model with *J* pathways. Rectangles and circles represent observed variables (mRNA expression) and latent variables (pathways), respectively. Each pathway consists of three or more genes and is represented by a latent variable constructed by a weighted sum of its genes. Single-headed arrows represent the effect of genes in a pathway, and the effect of pathways on the hazard function at the survival time *y*.

Let *y_i_* denote a survival time (*i* = *1*,…,*I*). Let *x_ijk_* denote the *kth* gene (*k* = *1*,…,*m_1_*,…,*K*) expression corresponding to the *jth* pathway (*j* = *1*,…,*J*) for the *ith* patient. As shown in Figure 1, we must then consider latent structures for estimating the model parameters. Let *w_jk_* denote the weight assigned to *x_ijk_*. The coefficient *β_j_* represents the effect of the latent variable *f_ij_* on the phenotype. *Wj* = [*w_j1_*,…,*w_jmj_*]’, *B* = [*β_1_*,…,*β_j_*]’. Considering this structure, we designed the following Cox proportional hazard model.

$$h\left(y_{i}{|f}_{i}\right){\;=\;h}_{0}{(y}_{i})\exp(\sum_{j=1}^{J}(\sum_{k=1}^{m_{j}}x_{\mathrm{ijk}}w_{\mathrm{jk}}{)\beta }_{j}{)\;=\;h}_{0}{(y}_{i})\exp(\;\sum_{j=1}^{J}f_{\mathrm{ij}}\beta_{j})$$

To estimate the model parameters for HisCoM-PAGE, we maximized the penalized partial log likelihood using a ridge penalty. The following Equation (1) then represents the objective function, which is a partial log likelihood from a Cox model.

(1) $$\begin{array}{l} \phi \;=\underset{{i:C}_{i}=1}{\sum }(\sum_{j=1}^{J}f_{\mathrm{ij}}\beta_{j}-log\underset{l\in {R(y}_{i})}{\sum }\exp(\sum_{j=1}^{J}f_{\mathrm{lj}}\beta_{j}))\\ \;-\frac{1}{2}\lambda_{\mathrm{gene}}\sum_{j=1}^{J}\sum_{k=1}^{m_{j}}P_{\lambda_{\mathrm{pathway}}}\left(w_{\mathrm{jk}}\right)-\frac{1}{2}\lambda_{\mathrm{pathway}}\sum_{j=1}^{J}P_{\lambda_{\mathrm{gene}}}\left(\beta_{j}\right)\;,\;where\;f_{\mathrm{ij}}=\;\sum_{k=1}^{m_{j}}x_{\mathrm{ijk}}w_{\mathrm{jk}} \end{array}$$

Here *y_1_ < y_2_ <…<y_d_* denote the distinct and ordered d(≤I) survival times and R(*y_i_*) is the risk set at time *y_i_* The objective function can be maximized by an alternating least squares (ALS) algorithm, which iterates the following two steps until convergence. In the first step, the pathway coefficients are estimated and updated, using a least-squares approach. For the second step, the weight coefficients are updated for fixed-path coefficient estimates [31]. In HisCoM-PAGE, we adopted a ridge penalty to address the multicollinearity of genes. When estimating *λ_gene_* and *λ_pathway_* values, we conducted 5-fold cross-validation to obtain optimal values for *λ_gene_* and *λ_pathway_* The process of estimating the coefficients, using the ALS algorithm, with penalty, proceeds as follows:

$$\mathrm{Let}\;\eta \;=\;\mathrm{XWB},\;u\;=\;\frac{\partial \phi }{\partial \eta }\;,\;A\;=\;\frac{\partial^{2}\phi }{\partial {\eta \eta }^{T}}{,\;z\;=\;\eta +A}^{-1}u,$$

1. Initialize $\hat{\;B\;}\;,\;\hat{W\;}=\;0.$
2. Compute η, u, A, and z, based on the latest value of $\hat{\;B\;}\;,\;\hat{W\;}$. Then maximize $\phi \left(W,B\right)$ with the fixed $\hat{W\;}.$ Repeat these steps until $\hat{\;B\;}$ converges.
3. Compute η, u, A, and z, based on the latest value of $\hat{\;B\;}\;,\;\hat{W\;}$. Then maximize the $\phi \left(W,B\right),$ with the updated $\hat{\;B\;}$. Repeat these steps until $\hat{W\;}$ converges.
4. Iterate steps 2 and 3 until $\phi \left(W,B\right)$ converges.

As a convergence criterion, we used the difference between ϕ(W, B) of consecutive iterations. The iteration continues until this difference is smaller than the given threshold value. In our analysis, the threshold value was 10^−4^. Since it is not straightforward to obtain asymptotic variance estimates of parameters from ridge estimation, we used a permutation test to obtain a statistical significance level for HisCoM-PAGE.

#### 2.2.2. Comparison Methods

The following pathway methods were considered to compare the results of HisCoM-PAGE. We compared other pathway methods such as Gene Set Enrichment Analysis (GSEA) [12], Global Test (GT) [15,16] and the Wald type test [17]. The GSEA methods assume that the total number of genes *K* and the gene set *S* is predefined. For the first step, compute the regression coefficients of *K* genes, by fitting a univariate Cox models. The regression coefficient is used as an association measure between phenotype and genes. Secondly, order *K* genes by the absolute value of t statistics in descending order. Thirdly, calculate the enrichment score. While computing the enrichment score, GSEA method consider two methods of weighting, including GSEA1, the case when the weight term is 0, and GSEA2, the modified version of the original GSEA method when the weight term is 1 [12,29]. Lastly, calculate the significance level by comparing the observed values and the permutation distribution values. The Global test is based on the regression coefficients from a Cox model. The Global tests can test whether the expression of gene, within a predefined pathway, tends to closely associate with the survival times. The global test’s Q statistic was taken as an average of the *m* test statistics calculated from each *m* individual gene, constituting a pathway by itself. Although the *p*-values can be calculated using the permutation and asymptotic method, we used the permutation approach. Thirdly, the Wald type test is based on the unified pathway method proposed which combined component-wise test statistics for significance of a subset of genes [17]. The Wald test also assesses whether the predefined pathway has an association with survival times. Thus, the test statistic is a sum of squares of the Wald statistic for the individual genes that constitute the pathway.

## 3. Results

### 3.1. Real Data Analysis Result

#### 3.1.1. Pathway Analysis Using SNUH Microarray Data

The Affymetrix gene identifiers were mapped to the Kyoto Encyclopedia of Genes and Genomes (KEGG) and Biocarta databases [32,33]. For the KEGG database, 4,320 genes were mapped to 185 pathways including overlapping genes. For the Biocarta database, 4,317 genes were mapped to 216 pathways including overlapping genes. Our objective in this pathway analysis was to identify pathways associated with PDAC patients’ overall survival times.

For multiple test adjustment, we used the False Discovery Rate (FDR) analysis to calculate the FDR adjusted *q*-value as a criterion [34]. From the Biocarta database, HisCoM-PAGE identified four significant pathways with FDR-adjusted *q*-value smaller than 0.05. The upper part of Table 2 shows the list of significant pathways related to the survival times. The transforming growth factor β (TGF-β) pathway was found to be the most significant. It is well known that the TGF-β pathway is associated with inflammation promotion and carcinogenesis in the early stage of cancer [35,36,37,38,39,40,41]. Transducer of ERBB2 (TOB1) has previously been reported to be linked to PDAC [42,43].

From the KEGG database, HisCoM-PAGE identified 23 pathways with FDR-adjusted *q*-values smaller than 0.05. The lower part of Table 2 shows the list of significant pathways related to the survival times. 

The pathways reported to be related to PDAC or pancreatic cancer are as follows. Hedgehog-signaling dysregulation, due to mutation or overexpression of pathway components and pathway ligands, induces pancreatic cancer [44]. The Wnt signaling pathway is related to drug resistance of pancreatic cancer and its function has an association with prognosis [45,46]. The Vascular Endothelial Growth Factor(VEGF) pathway is angiogenesis related [47], and the insulin-signaling pathway is studied for pancreatic cancer growth and metastasis [48]. Insulin receptor contributes to the Phosphatidylinositol-3-kinase (PI3K)-Akt pathway activation and this pathway mediates the therapeutic resistance [49]. The fatty acid metabolism pathway is closely related to tumor cell survival and growth [50,51]. Glycosphingolipids of the ganglio globo-series pathway have been reported as carbohydrate antigens with cancer [52]. The steroid hormone synthesis pathway has been studied for PDAC. In pancreatic cancer cells, cholesterol can be converted into oxysterol or can be used as a precursor for steroid hormone synthesis [53]. The glycerophospholipid metabolism pathway is significant for PDAC progression [54]. The mammalian TOR (MTOR) signaling pathway is reported to be related to metastasis of PDAC [55]. The ErbB signaling pathway is a mediator or has tumor stroma interactions in PDAC [56].

**Figure 2:**
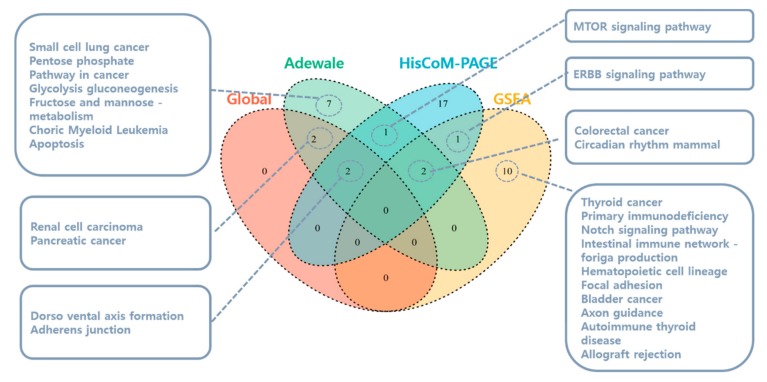
Venn diagram for the significant pathways identified by four different methods using the KEGG database. The pathways are listed with FDR-adjusted *q*-values less than 0.05. The 17 pathways uniquely identified by HisCoM-PAGE are highlighted in Table 2.

Figure 2 is a Venn diagram showing the numbers of pathways and their list commonly identified by HisCoM-PAGE and other methods. Each pathway-based method seems to provide a different list of significant pathways. No pathway was identified by all four methods. The four pathways selected significantly by three methods are as follows: Adherens junction pathway, Colorectal cancer pathway, Circadian rhythm mammal pathway, and Dorso vental axis formation pathway. Among these pathways, only the E-cadherins protein in the Adherens junction pathway is reported to have an association with lymph node metastasis in high-grade PDAC [57,58].

#### 3.1.2. Pathway Analysis Using TCGA RNA-Seq Data 

We performed RNA-seq data analysis for a replication purpose of SNUH microarray analysis results. Thus, only the genes used for SNUH microarray data analysis were selected for RNA-seq analysis. As a result, for the KEGG database, 3,258 genes were mapped to 185 pathways. For the Biocarta database, 1,162 genes were mapped to 216 pathways. 

Table 2 shows the RNA-seq data results. The last three columns summarize the results of RNA-seq data. Since RNA-seq analysis was performed only for a replication purpose, FDR was used to correct the significant pathways identified from SNUH microarray data analysis. There were no significant pathways with FDR-adjusted *q*-value smaller than 0.05. From the Biocarta database, TGF-β pathway’s nominal *p*-value was 0.053. For the KEGG database, the Amyotrophic lateral sclerosis pathway and MTOR signaling pathway were significantly identified with a nominal *p*-value Less than 0.05. 

In addition, among the pathways significant at the nominal level by RNA-seq analysis, the Neurotrophic signaling pathway showed the FDR-adjusted *q*-value close to 0.05 by microarray data analysis (*p*-value = 0.007 and FDR-adjusted *q*-value = 0.052). The neurotrophic signaling pathway is known to promote pancreatic cancer invasion [59].

#### 3.1.3. PDAC-Related Genes Using SNUH Microarray Data

With pathways associated with prognosis, we could also find genes meaningfully related to PDAC prognosis, as well as considering hierarchies of genes and pathways. Table 3 shows genes significant for the survival phenotype. Using the coefficients of our proposed model, we were able to calculate the *w_gene_ × β_path_* value for each gene. As a result, it was possible to simultaneously consider the effect of the matched gene to the pathway, and the effect size of the pathway to the phenotype. After calculating each coefficient, significance testing was performed, using a permutation method. If the marker was selected based only on a nominal *p*-value, obtained by adapting the entire gene, a type 2 error and the false negative error can be large. Therefore, we used FDR analysis to calculate the FDR-adjusted *q*-value as a criterion.

Table 3 shows the significant genes with FDR-adjusted *q*-values < 0.05. *SMAD3, BCL2*, and *TGF-β1* are well known to be related to PDAC. Upregulated *SMAD3* promotes epithelial mesenchymal transition and predicts poor PDAC [60,61]. Also, it has been reported that *BCL2* downregulated expression is a poor prognostic factor for PDAC [62]. *TGF-β1* levels are significantly related to PDAC patients’ prognosis [63]. That is, the patients with high levels of *TGF-β1* showed higher overall survival times. Some other PDAC-related genes are as follows. *ETS1* is known to have resistance to pancreatic cancer chemotherapy. Furthermore, *ETS1* can exacerbate poor PDAC prognosis after radiation therapy [64]. *HIF1A* was reported as a significant indicator of PDAC prognosis [65,66,67]. *GNAI1* is known to be a suppressor of tumor cell migration and invasion that is post-transcriptionally targeted by *mir-320a/c/d* [68]. *Mir-320a* is found to confer 5-FU chemo-resistance upon human pancreatic cancer cells [69].

#### 3.1.4. PDAC-related Genes Using TCGA RNA-Seq Data 

Table 3 shows the gene results from the TCGA RNA-seq data analysis. FDR was used for significant genes identified from SNUH microarray data analysis. For the Biocarta database, *GNAI1* was also significant with a *p*-value less than 0.05, and *BCL2L1* was significant with a *p*-value less than 0.1. In addition, the *p*-value of *SMAD3* was close to 0.1. However, *TGF-β1* and *ETS1* were not replicated. For the KEGG database, the *p*-value of *SMAD3* was less than 0.1.

### 3.2. Simulation Analysis Result

#### 3.2.1. Type 1 Error

**Figure 3:**
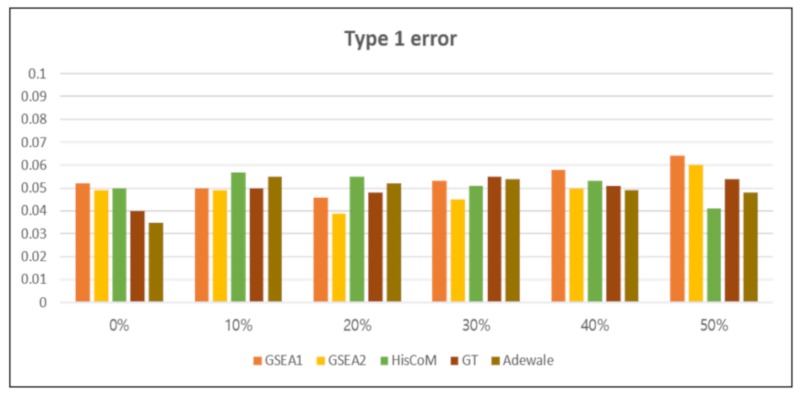
The empirical type 1 error with 1000 replicates at the 0 to 0.5 censoring proportions. The x-axis represents the censoring proportion and the y- axis represents the type 1 error. Comparison methods are as follows: Gene Set Enrichment Analysis with weight zero (GSEA1), Gene Set Enrichment Analysis with weight 1 (GSEA2), HisCoM-PAGE (HisCoM), Global test (GT) and Wald type test (Adewale).

Figure 3 shows the simulation results, for each method, when total number of genes (K) = 200, sample size (*I*) = 80, gene set size (𝑚_𝑠_)= 50, and censoring proportion (*c_p_*) = 0, 0.1, 0.2, 0.3, 0.4, 0.5. The number of permutations for significance testing was 1000. We checked the 95% confidence interval level of the estimated type 1 error. Overall, type 1 errors were shown to be well controlled in various scenario settings; especially in the HisCoM-PAGE method, the type 1 error is well controlled, even when the censoring fraction is high.

#### 3.2.2. Comparison of Power

**Figure 4:**
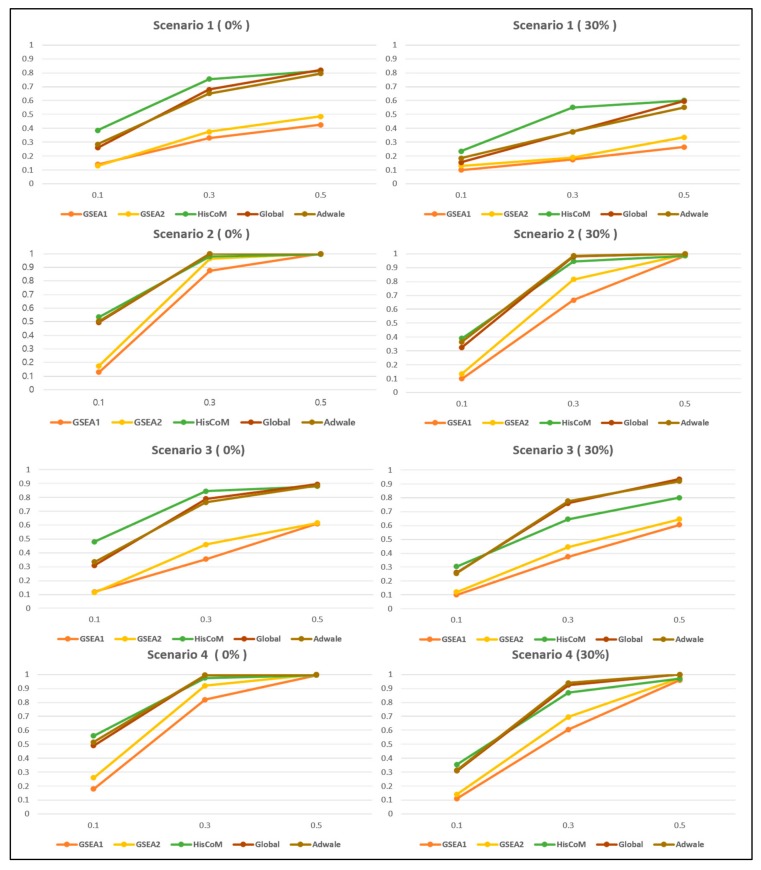
Empirical power of four scenarios. For the simulated gene expression data set, four correlation structures were considered. The x-axis refers to significant gene proportion, and the y-axis represents power. The percentage in the parenthesis indicates the censoring proportion.

For power analysis, we varied the censoring proportion and the proportion of significant genes in the causal pathway. We set the parameters as follows: total genes (*K*) = 200 sample size (*I*) = 80, pathway size (*m_s_*) = 50, significant gene proportion (*m_p_*) = 0.1, 0.3, 0.5 and censoring proportion (*c_p_*) = 0, 0.3. HisCoM-PAGE showed better performance than the other methods, when the significant gene proportions were not high, and the power was close to 1, when the significant gene proportion increases. Figure 4 shows the power of each method for the four correlation structure scenarios. Overall, the Global and the Wald type tests showed similar trend in power, and GSEA showed a relatively low power, compared to other methods, in many scenarios, as shown in the paper [29]. As shown by Chiristiaan [70], power depends largely on gene proportion, which has effects within a causal pathway. In Scenario 1, when all gene expression values are independent of each other and compared to other scenarios, the statistical power is strongly affected by the centering ratio. In Scenario 2, when the correlation coefficient between casual genes has the same effect, we could see a relatively high power compared to other scenarios.

For the GSEA method, the Cox model was only used for ordering genes, but enrichment scores were calculated using the relative rank only. In the case of the competitive analysis of the pathway methodology, observed statistics of the pathway of interest are compared with those of the pathway consisting of the genes within the pathway [71]. By contrast to GSEA-based method, HisCoM-PAGE can directly calculate the effect of the causal pathway, quantitatively, on the survival time. We could also confirm that the power of HisCoM-PAGE was higher than the other methods in Scenario 1, even when the censoring ratio was high. 

## 4. Discussion and Conclusions

Among pathway analysis methods, few have been developed only for survival times. Thus, there is a need to quantitatively determine how much the pathway affects the survival phenotype and to identify a relative way of ranking pathways. To this end, HisCoM-PAGE uses structural equations to model real biological phenomena, so it can estimate not only the value of statistics of a pathway but also the meaning of pathway statistics. In other words, HisCoM-PAGE can find not only pathways related to prognosis but also how strongly genes contribute to the pathway. The estimated weight represents the gene effect on the pathway, and *β_path_* represents the effect of pathway on the hazard rate through a Cox model.

It is well known that biological phenomenon is a result of complex pathway interactions. That is, multiple pathway analysis provides a more biologically interpretable result than single pathway analysis. HisCoM-PAGE can perform multiple pathway analysis by considering all pathways simultaneously. No existing pathway methods can perform these analyses.

Recently, many studies have been actively conducted to examine cancer prognosis using RNA-seq data [26,72]. HisCoM-PAGE could easily be applicable to RNA-seq data. From the SNUH microarray data, HisCoM-PAGE identified PDAC prognosis-related pathways such as TGF-β, Hedgehog signaling pathways, MTOR signaling pathway, and so on. As a pilot study, we conducted the replication analysis using TCGA RNA-seq data. Application of HisCoM-PAGE to the TCGA RNA-seq data replicated TGF-β and MTOR signaling pathways. Furthermore, HisCoM-PAGE also replicated the *GNAI1* gene as a PDAC prognosis-related gene among the genes *SMAD3, BCL2, TGF-β1* and *GNAI1* identified from SNUH microarray data analysis. 

There have been several studies for comparing microarray and RNA-seq platforms [73,74]. For example, in identifying differentially expressed genes (DEGs), there were a number of genes specifically detected as DEGs in only one platform, while a large number of genes were identified as DEGs in both microarray data and RNA-seq data platforms [74]. A large discrepancy is expected between the two platforms when the treatment effect is small. In our pathway analyses, there was a discrepancy in analysis results between microarray data and RNA-seq data. To find out the main reason for this discrepancy, first the expression profiles of genes in two platforms were compared. The correlation between the averages of normalized and log2 transformed values of two platforms was high, with Pearson correlation coefficient of 0.62 and Spearman correlation coefficient of 0.72. However, when the *p*-value from a univariate Cox model for each gene was compared between the two platforms, the correlation of −*log*(*p*-value)*s* was low, with Pearson correlation coefficient of 0.25 and Spearman correlation coefficient of 0.23. This low correlation was caused by the different survival curves between microarray data and RNA-seq data. The two Kaplan-Meier curves from both microarray data and RNA-seq data were quite different and the log-rank test for the equivalence for these two survival curves has a *p*-value of 0.046 [75]. Since HisCoM-PAGE is based on the association between the survival time and genes based on a Cox model, it might be that the discrepancy of the pathway analyses between microarray and RNA-seq data was caused by the heterogeneity of survival times between two platforms. It could be expected that the samples with less heterogeneous survival times could provide more consistent results.

Next, we are planning to improve the performance of HisCoM-PAGE by taking the characteristics of RNA-seq data into account. Then, we need to conduct a simulation study to investigate its properties and compare it with other methods for RNA-seq data. 

We believe that HisCoM-PAGE can be easily applicable to other types of omics data such as integration analysis of multi-omics data. Furthermore, we can also use the HisCoM-PAGE approach to construct predictive models for prognosis. We could study a design of a prognostic prediction model using the latent variable pathway as a marker, as well as the genetic marker [76]. In this case, unlike building predictive models using only genetic markers, we can add interpretability because the designed predictive model considers the contribution of genetic markers in a pathway manner. Also, it would be possible to study the relationship between genes and pathways, beyond the linear relationship, using the kernel generalized structured component analysis (GSCA) method. 

In summary, we proposed a new pathway analysis method, Hierarchical Structural Component Models of Pathway Analysis for Gene Expression (HisCoM-PAGE), to identify disease prognosis-related pathways. Using simulated data, PDAC microarray and RNA-seq data, we can confirm that HisCoM-PAGE can identify pathways and genes that have an association with a survival phenotype. Moreover, HisCoM-PAGE can also find interpretable and meaningful pathways and prognostic genetic markers. 

## Figures and Tables

**Table 1:** Demographics and clinical characteristics of study patients.

Variable	Variable Description	Descriptive Statistics
Age	Age at diagnosis	63.32(10.064) mean(se)
Sex		Male: 75, Female: 50
Positive Lymph Nodes (LN)	Number of cancers transmitted by Lymphocytes	(0,1,2) (1st Quantile, Median,3rd Quantile)
Size	Maximum Tumor Size (cm)	3.574 (mean)
Differentiation	Clinico-pathologic characteristics and prognostic value of various histological types.	WD: 19, MD: 85, PD: 18, Other: 2 (NA: 1)
Jaundice		Yes: 89, No: 36
7th staging T stage	AJCC 7th T staging criteria. The extent of the tumor.	1th: 6, 2nd: 3, 3rd: 104, 4th: 12
7th staging N stage	AJCC 7th N staging criteria. The spread to nearby lymph nodes.	Yes: 71, No: 54
Radiation therapy	Radiation therapy after surgery	Yes: 72, No: 53
Chemotherapy	Chemotherapy after surgery	Yes: 94, No: 31
Overall survival time		Median: 25 months

Differentiation variables were constituted the following categories: Well Differentiated (WD), Moderately Differentiated (MD) and Poorly Differentiated (PD). For T and N stage, American Joint Committee on Cancer (AJCC) 7th edition was applied.

**Table 2:** Significant pathways for PDAC prognosis identified by HisCoM-PAGE using SNUH microarray data and the replication study result using TCGA RNA-seq data.

Pathway Database	Pathway Name	Microarray Data			RNA-seq Data		
		|β_path_|	*p*-Value	*q*-Value	|β_path_|	*p*-Value	*q*-Value
BIOCARTA	Transforming Growth Factor-β (TGF-β)*	0.017	0.00001	0.002	0.006	0.053	0.211
	Non-Typeable Haemophilus Influenzae (NTHI)	0.014	0.00033	0.03	0.006	0.314	0.419
	MITOCHONDRIA	0.01	0.00054	0.03	0.007	0.197	0.394
	Transducer Of ERBB2.1(TOB1)*	0.016	0.00056	0.03	0.005	0.46	0.46
KEGG	**BASAL CELL CARCINOMA**	0.015	0.0001	0.0074	0.0071	0.261	0.894
	COLORECTAL CANCER	0.014	0.0001	0.0074	0.0085	0.843	0.959
	CIRCADIAN RHYTHM MAMMAL	0.01	0.0012	0.0306	0.0062	0.869	0.959
	FATTY ACID METABOLISM*	0.012	0.0009	0.0306	0.0088	0.868	0.959
	GLYCOSPHINGOLIPID BIOSYNTHESIS GLOBO SERIES*	0.01	0.0008	0.0306	0.0018	0.364	0.9055
	INSULIN SIGNALING PATHWAY*	0.016	0.0011	0.0306	0.013	0.951	0.959
	STEROID HORMONE BIOSYNTHESIS*	0.014	0.0015	0.0306	0.0037	0.541	0.959
	Vascular Endothelial Growth Factor (VEGF) SIGNALING *	0.014	0.0007	0.0306	0.0078	0.724	0.959
	WNT SIGNALING*	0.017	0.0014	0.0306	0.0104	0.415	0.905
	ADHERENS JUNCTION*	0.013	0.0018	0.0326	0.01	0.819	0.959
	**DILATED CARDIOMYOPATHY**	0.016	0.0023	0.0326	0.011	0.851	0.959
	**OTHER GLYCAN DEGRADATION**	0.009	0.0021	0.0326	0.0031	0.665	0.959
	OXIDATIVE PHOSPHORYLATION*	0.015	0.0022	0.0326	0.0099	0.178	0.894
	**AMYOTROPHIC LATERAL SCLEROSIS(ALS)**	0.01	0.0028	0.0369	0.0108	0.004	0.0852
	DORSO VENTRAL AXIS FORMATION	0.009	0.0031	0.0369	0.0036	0.344	0.905
	**SULFUR METABOLISM**	0.008	0.0032	0.0369	0.0046	0.212	0.894
	**CARDIAC MUSCLE CONTRACTION**	0.013	0.0038	0.0373	0.0086	0.732	0.959
	ERBB SIGNALING*	0.013	0.004	0.0373	0.0069	0.401	0.9055
	GLYCEROPHOSPHOLIPID METABOLISM*	0.015	0.0036	0.0373	0.0121	0.952	0.959
	**HYPERTROPHIC CARDIOMYOPATHY (HCM)**	0.015	0.004	0.0373	0.0127	0.959	0.959
	**GLIOMA**	0.012	0.0044	0.039	0.0081	0.711	0.959
	Mammalian TOR (MTOR) SIGNALING*	0.011	0.0055	0.0465	0.0098	0.014	0.112
	HEDGEHOG SIGNALING *	0.011	0.0058	0.047	0.0068	0.258	0.894

Pathways related to PDAC or pancreatic cancer are denoted by *. The pathways uniquely identified by HisCoM-PAGE are denoted as bold. Kyoto Encyclopedia of Genes and Genomes (KEGG).

**Table 3:** Significant genes in PDAC prognosis identified by HisCoM-PAGE using the SNUH microarray dataset and the replication study result using the TCGA RNA-seq data.

Pathway Database	Pathway Name	Gene	SNUH Microarray			TCGA RNA-seq		
			|w_gene_ × β_path_|	*p*-Value	*q*-Value	|w_gene_ × β_path_|	*p*-Value	*q*-Value
BIOCARTA	Non-Typeable Haemophilus Influenzae (**NTHI**)	*SMAD3*	0.032	0.00001	0.004	0.004	0.1246	0.298
	Transducer Of ERBB2.1(**TOB1**)	*SMAD3*	0.032	0.00001	0.004	0.004	0.1134	0.298
	Transforming Growth Factor-β (**TGF-β**)	*SMAD3*	0.032	0.00001	0.004	0.004	0.1114	0.298
	CHEMICAL	*BCL2L1*	0.024	0.00003	0.004	0.006	0.0762	0.259
	IL-2 receptor beta chain (IL2RB)	*BCL2L1*	0.024	0.00003	0.004	0.006	0.0866	0.266
	RAS	*BCL2L1*	0.024	0.00003	0.004	0.006	0.0783	0.259
	Bcl-2 antagonist of cell death (BAD)	*BCL2L1*	0.024	0.00003	0.004	0.006	0.0777	0.259
	**MITOCHONDRIA**	*BCL2L1*	0.024	0.00003	0.004	0.006	0.0753	0.259
	CCCTC-binding factor (CTCF)	*TGF-β1*	0.019	0.00005	0.004	0.008	0.9707	0.982
	Inflammatory Response(INFLAM)	*TGF-β1*	0.019	0.00005	0.004	0.008	0.9715	0.982
	Erythrocyte Differentiation (ERYTH)	*TGF-β1*	0.019	0.00005	0.004	0.008	0.9716	0.982
	MAP Kinase(MAPK)	*TGF-β1*	0.019	0.00005	0.004	0.008	0.9726	0.982
	Anaplastic lymphoma kinase(ALK)	*TGF-β1*	0.018	0.00006	0.004	0.008	0.9695	0.982
	G1	*TGF-β1*	0.018	0.00006	0.004	0.008	0.9706	0.982
	P38MAPK	*TGF-β1*	0.019	0.00006	0.004	0.008	0.9718	0.982
	**TOB1**	*TGF-β1*	0.018	0.00006	0.004	0.008	0.971	0.982
	NKT	*TGF-β1*	0.018	0.00006	0.004	0.008	0.971	0.982
	Interleukin-1 receptor (IL1R)	*TGF-β1*	0.018	0.00006	0.004	0.008	0.971	0.982
	**TGF-** **β**	*TGF-β1*	0.018	0.00006	0.004	0.008	0.971	0.982
	KERATINOCYTE	*ETS1*	0.015	0.00008	0.005	0.001	0.3516	0.643
	E-26-specific (ETS)	*ETS1*	0.015	0.0001	0.006	0.001	0.3588	0.643
	P53HYPOXIA	*HIF1A*	0.016	0.00047	0.028	0.0002	0.4766	0.762
	Hypoxia-Inducible Factor(HIF)	*HIF1A*	0.016	0.00047	0.028	0.0001	0.4767	0.762
	Erythropoietin mediated neuroprotection through NF-kB (EPONFKB)	*HIF1A*	0.016	0.0005	0.028	0.0001	0.4786	0.762
	Vascular Endothelial Growth Factor (VEGF)	*HIF1A*	0.015	0.0006	0.033	0.00005	0.9824	0.982
	DEATH	*TNFRSF10B*	0.018	0.00064	0.033	0.002	0.647	0.897
	Formyl methionyl leucyl phenilalanine (FMLP)	*GNA15*	0.015	0.00074	0.037	0.006	0.0485	0.24
	IL1R	*IL1RAP*	0.01	0.00095	0.041	0.002	0.2839	0.581
	SET	*GZMA*	0.015	0.001	0.041	-	-	-
	Phosphoinositides (PTDINS)	*PFKP*	0.011	0.0011	0.041	0.00008	0.5064	0.778
	Extrinsic Prothrombin Activation (EXTRINSIC)	*TFPI*	0.013	0.00115	0.041	0.002	0.6457	0.897
	Acute Myocardial Infarction (AMI)	*TFPI*	0.013	0.00116	0.041	0.002	0.6457	0.897
	protease-activated receptors-1 (PAR1)	*GNAI1*	0.017	0.00118	0.041	0.007	0.0502	0.24
	Endothelial differentiation gene- 1 (EDG1)	*GNAI1*	0.017	0.00119	0.041	0.007	0.0464	0.24
	G protein-coupled receptors (GPCR)	*GNAI1*	0.017	0.00119	0.041	0.007	0.0499	0.24
	SPPA	*GNAI1*	0.017	0.00122	0.041	0.007	0.0481	0.24
	Bioactive Peptide Induced Signaling (BIOPEPTIDES)	*GNAI1*	0.017	0.00122	0.041	0.007	0.0476	0.24
	CXC chemokine receptor type-4 (CXCR4)	*GNAI1*	0.017	0.00122	0.041	0.007	0.0447	0.24
	Mannose 6-phosphate receptors (MPR)	*GNAI1*	0.017	0.00122	0.041	0.008	0.0432	0.24
	Glycogen synthase kinase-3 (GSK3)	*GNAI1*	0.017	0.00123	0.041	0.008	0.0432	0.24
	Peroxisome proliferator-activated receptor alpha (PPARA)	*ACOX1*	0.015	0.00122	0.041	0.003	0.3221	0.63
	VEGF	*VEGFA*	0.01	0.00146	0.047	0.003	0.1576	0.339
	Nitric Oxide-1(NO1)	*VEGFA*	0.01	0.00147	0.047	0.003	0.1575	0.339
KEGG	CELL CYCLE	*SMAD3*	0.023	0.0001	0.047	0.003	0.099	0.099
	**WNT SIGNALING**	*SMAD3*	0.023	0.0001	0.047	0.003	0.096	0.099
	TGF-β	*SMAD3*	0.023	0.0001	0.047	0.003	0.0957	0.099

Bold pathways were significantly identified by HisCoM-PAGE.
